# Bristol Rabbit Pain Scale (BRPS): clinical utility, validity and reliability

**DOI:** 10.1186/s12917-022-03434-x

**Published:** 2022-09-09

**Authors:** L. Benato, J. Murrell, N. Rooney

**Affiliations:** 1 Animal Welfare and Behaviour, School of Veterinary Sciences, University of Bristol, Langford, UK; 2 Highcroft Veterinary Referrals, 615 Wells Road, Whitchurch, Bristol, BS14 9BE UK

**Keywords:** Rabbit, Pain, Pain scale, Validity, Reliability, Clincal utility

## Abstract

**Background:**

The Bristol Rabbit Pain Scale (BRPS) was developed using a combination of methods, focus groups and behavioural observation, that led to a composite pain scale of six categories (Demeanour, Locomotion, Posture, Ears, Eyes and Grooming) with four intensities of pain (0, 1, 2, and 3), and a total score of 0–18. The aim of this study was to assess the clinical utility, validity and reliability of the BRPS.

**Materials and methods:**

The clinical utility of the BRPS was tested using a questionnaire composed of ten questions each on a five-point Likert scale ranging from one (strongly disagree) to five (strongly agree). The respondents, (veterinary surgeons and veterinary nurses), were asked to assess up to four rabbits in acute pain, using the novel pain. They then completed the questionnaire which asked whether the BRPS was easy and quick to use and whether it provided information that was clinically useful. The questionnaire was tested for internal reliability using the Cronbach’s alpha reliability coefficient. The construct validity (how well the tool measures the concept it was designed for) was measured by observers blindly rating 20 rabbits pre- and post-surgery whilst the criterion validity (the degree to which the tool correlates with a gold standard) was assessed by correlating BRPS scores with scores using a numerical rating scale (NRS) with a total score of 0–10. Inter-rater reliability was tested by quantifying the agreement in the pain scores given by nine participants when assessing the same 40 video clips. The intra-rater reliability was measured by testing how consistent the participants were when rating the same clips one month later.

**Results:**

The median score of the ten questions of the clinical utility test was 4 (range 2–5). The Cronbach’s alpha reliability coefficient of the clinical utility test was good (α = 0.811) demonstrating good internal consistency. The median (range) pain score of the BRPS and the NRS were 3 (0–14) and 0 (0–8) before surgery and 12 (1–18) and 7 (0–10) after surgery respectively. The BRPS demonstrated high construct validity (Z = -11.452; *p* < 0.001) and there was a strong correlation between the BRPS and the NRS (Rho = 0.851; *p* < 0.001) indicating high criterion validity. The inter-rater and the intra-rater agreements were α = 0.863 and α = 0.861 respectively, which is considered good.

**Conclusions:**

This study showed that the BRPS is a suitable tool for quantifying pain in rabbits in a clinically useful, valid and reliable way.

## Background

Pain is a debilitating condition that affects not only people but also animals [1]. Recently significant focus has been given to the development of pain assessment tools for companion animals such as cats [2], dogs [3], and rabbits [4] with the aim of improving the health and wellbeing of the patient. A reliable pain assessment tool should be species-specific to the patient assessed [5] and should adequately identify and quantify the pain that the subject experiences [6]. Moreover, it should be practical and easy-to-use [7] as evidence shows that a lengthy and time-consuming tool is less likely to be applied in clinical practice [8].

Once a suitable tool is developed, it should be tested for its clinical utility, validity and reliability. A clinical utility test, defined as the “evaluation of clinical effectiveness’ of a novel tool [8], is generally carried out using a survey method that enables assessment of the new tool with the aim of improving aspects of the clinical routine.

The validity of a pain scale is defined as “the degree to which a test measures either a quantity or hypothetical construct which it is intended to assess” [6]. Various aspects of validity such as construct validity [9], and criterion validity can be assessed. Construct validity is tested to determine if a pain scale can detect changes in pain severity. Criterion validity is determined by comparing the “test” pain scale against a gold standard [10]; a tool that is universally recognised as the optimum standard within the field.

Reliability [9] describes the extent to which ratings using the pain tool are consistent over time (intra-rater reliability) [10] and are consistent when two or more observers use the tool (inter-rater reliability) [10]. Achieving high reliability is important to reduce the subjectivity of the scale and to confirm the reproducibility and accuracy of the scores.

In the last few years, two pain scales specific for rabbits have been validated: the Rabbit Grimace Scale (RbtGS) [4] based on facial indicators, and the CANCRS (a composite pain scale developed at the Centro Animali Non Convenzionali, (C.A.N.C., Italy) [4] that merges the RbtGS with a clinical pain scale (CPS) measuring physiological parameters and behavioural responses. Although both scales are invaluable assets in the field of rabbit medicine, they present some limitations. The RbtGS is mainly designed for laboratory rabbits with straight ears while the CPS parameters of the CANCRS are based on pain scales developed for cats and dogs. We therefore developed a novel multidimensional pain scale, the Bristol Rabbit Pain Scale (BRPS) [11] (Table 1), which sought to overcome these limitations.

**Table 1:**
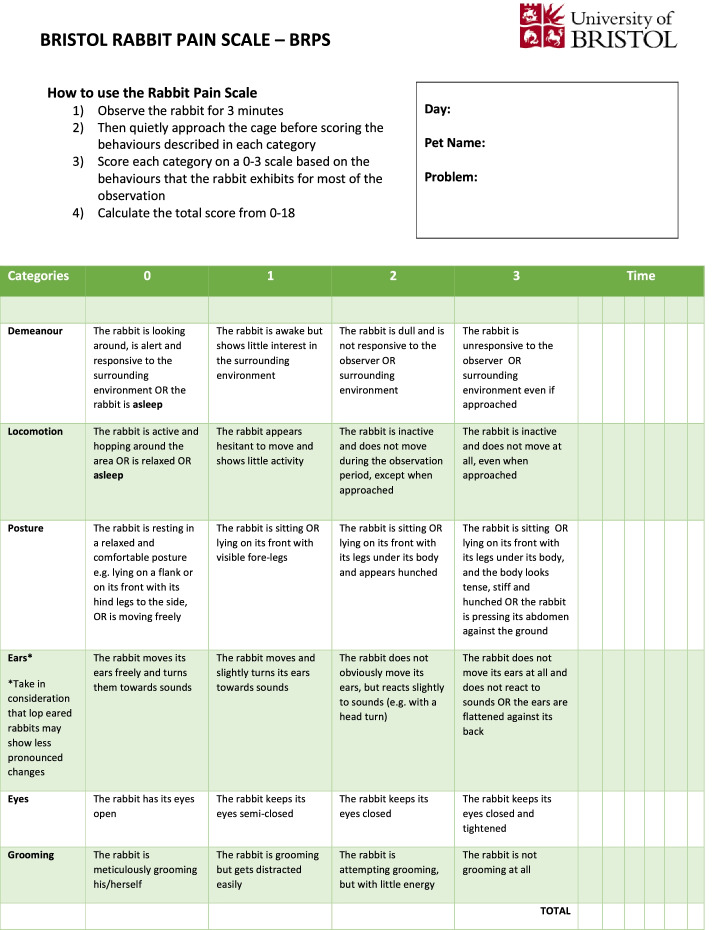
The Bristol Rabbit Pain Scale (BRPS) consists of six categories (Demeanour, Locomotion, Posture, Ears, Eyes and Grooming) with four intensities of pain (0, 1, 2, and 3), and a total score of 0-18. For a downloadable version see https://www.bristol.ac.uk/vet-school/research/projects/bristol-rabbit-pain-scale/

The BRPS consists of six categories (Demeanour, Locomotion, Posture, Ears, Eyes and Grooming) each with four intensities of pain (0, 1, 2, and 3), and produces a total score of 0–18. It was developed using a combination of methods: focus group discussion and behavioural observation of rabbits. A focus group discussion was carried out with stakeholders and specialist veterinary surgeons to assess the content validity of the novel scale. Behavioural observations of rabbits during the peri-operative period were carried out to confirm the elements of the scale specific to rabbits with and without pain. The BRPS is based on parameters that are specific to rabbits and allows the assessment of pain of both straight and lop-eared rabbits [11].

The aim of the current study was to assess the clinical utility, the construct and criterion validity and the reliability of the BRPS. A questionnaire was used to investigate the clinical utility. This was a modified version of the utility scale used previously in the analysis of pain assessment tools for children unable to self-report pain due to cognitive impairment [12] or young age [13]. We surveyed to what extent veterinary staff such as veterinary surgeons and veterinary nurses who had trialled the tool agreed with the statements such as the BRPS is a practical and easy-to-use pain tool and that it provides useful information for the clinician. The internal consistency of the questionnaire was tested using the Cronbach’s alpha reliability coefficient [14].

We hypothesised that there would be a statistically significant difference between pre-and post-surgery in terms of pain scoring (construct validity) and that there would be a correlation between the BRPS and a numerical rating scale (NRS) with a total score from 0–10 (where 0 is no pain and 10 is the most severe pain) defined as gold standard for the purposes of this study (criterion validity).

We also hypothesised that there would be agreement in the pain scores given by the multiple participants when assessing the same animals (inter-rater reliability), and that the participants would be consistent in their pain scores when repeatedly over time (intra-rater reliability). To test this, we used 40 videos of rabbits in varying degrees of pain which were rated by nine participants.

## Results

### Clinical utility test

A sample of 21 respondents (Female (F) = 18; Male (M) = 2; Unknown = 1) from veterinary clinics in the UK used the novel BRPS to assess a total of 58 rabbits in acute pain (Median age 3 y, range = 3 months -12y; M = 26; F = 24; Unknown = 8; straight eared = 21, lop eared = 34, one lop ear and one straight ear = 3). Each respondent rated between 1 and 4 rabbits. The median time reported to pain score the rabbits using the BRPS was 4 min (range 2–6 min). The median score of the ten questions was 4 (range 2–5; Table 2). The Cronbach’s alpha reliability coefficient for the clinical utility test was good (α = 0.811) demonstrating good internal consistency. Overall, the respondents reported finding the BRPS quick and easy to apply, that it provided information that was clinically useful, and that it could be incorporated into routine practice.

**Table 2:** Results of the clinical utility tests reported as median and range. The questions used a five-point Likert scale ranging from one (strongly disagree) to five (strongly agree)

	Median	Minimum	Maximum
It is easy and clear to understand	5	3	5
It is quick to apply	5	3	5
It is easy to apply	5	3	5
The time taken to complete the scale is appropriate for incorporation into routine practice	5	3	5
It discriminates rabbits with pain from rabbits without pain	4	3	5
This tool is better for pain assessment in rabbits than other currently available pain assessment tools	4	2	5
This tool is easier to use than my current method to assess pain in rabbits	5	2	5
It provides information that is clinically useful	4	3	5
Pain scoring using this tool can easily guide analgesic intervention	4	3	5
I would be happy to use this tool	5	2	5

The majority of the comments written by the respondents were supportive of the use of the BRPS such as: ‘I found it [the BRPS] very useful”, “It is actually a very quick procedure (to assess pain)”, “Much easier to use [than other pain scales]”, “Quite straightforward to use”. Some comments provided ideas for further development of the BRPS; for example: “[It is]Not clear as to action required”, “What score would prompt analgesia to be given?”.

### Validity

**Fig. 1:**
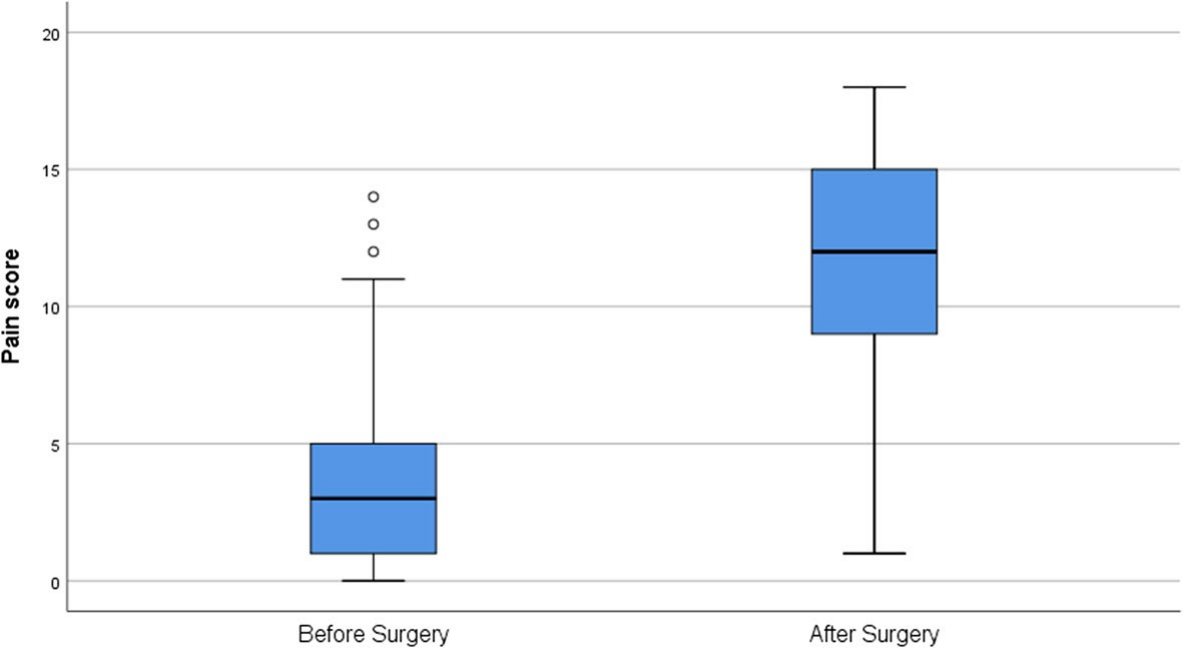
Box plot of median pain score given by the respondents before (3; range 0–14) and after surgery (12; range 1–18) using the BRPS showing significant difference between the two groups. The horizontal line denotes the median value (50th percentile), while the box contains the 25th to 75th percentiles. The upper and lower whiskers represent the greatest and least scores. ^○^ represents outsiders

**Fig. 2:**
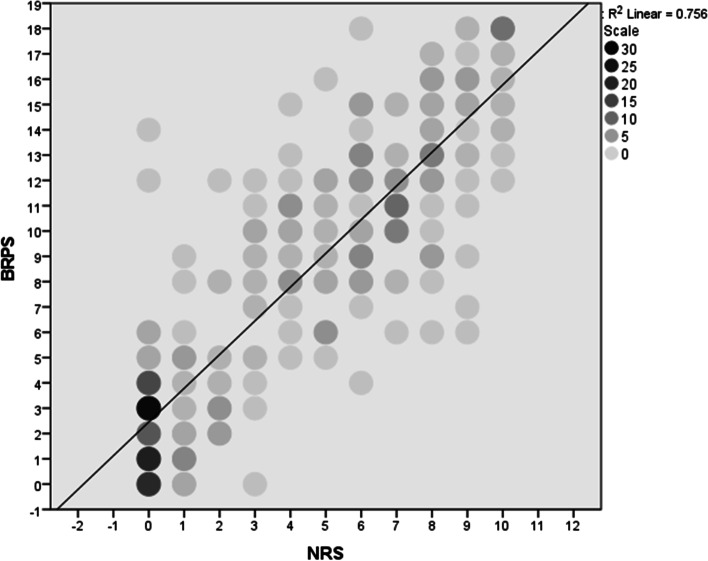
Scatterplot of the pain scores given by the participants scoring the video clips of the same 20 rabbits before and after surgery using the BRPS in relation to those given using the Numerical rating scale (NRS). The results show a positive correlation between the BRPS and the NRS. The colour of each dot represents the number of each data point

The median pain score (range) assigned by the participants using the BRPS before surgery was 3 (0–14) and after surgery was 12 (1–18). The median score (range) using the NRS was 0 (0–8) before surgery and 7 (0–10) after surgery. The Wilcoxon signed rank test demonstrated a statistically significant difference between the pre- and post-perioperative pain scores when using the BRPS (Z = -11.45; *p* < 0.001; Fig. 1). There was a strong correlation between the BRPS and the NRS (Rho = 0.851; *p* < 0.001; Fig. 2).

### Reliability

The inter-rater and the intra-rater agreements were good with Krippendorff’s α = 0.863 (LL95%CI 0.851- UL95%CI 0.874) and α = 0.861 (LL95%CI 0.783- UL95%CI 0.922) respectively.

## Discussion

In this study, we tested the clinical utility, validity and reliability of the previously developed BRPS. Each of these was found to be high, suggesting that the BRPS is a practical and valid pain assessment tool that can be used reliably to assess acute pain in rabbits in a clinical setting.

The clinical utility of the BRPS was demonstrated by gathering information from veterinary surgeons and veterinary nurses that had trailed the tool, using a questionnaire. The results showed that the BRPS has the potential to assist veterinary staff when evaluating and treating pain in rabbits.

The clinical utility of a pain tool is more commonly tested in human medicine either when developing a novel tool or when there is the need to compare several pain tools [12]. To the authors’ knowledge, this is the first time that a clinical utility test has been carried out to assess a novel pain instrument in veterinary medicine, although the utility of the Glasgow acute pain scale for cats has been inferred from users’ feedback [2]. It is unclear why clinical utility tests are not carried out in veterinary medicine. One reason could be that it is not considered as important as testing the reliability and validity and therefore it is generally overlooked, as is often also the case in human medicine [15]. A review of pain assessment tools for people with dementia, found that of a total of 28 tools for non-verbal patients only three had carried out specific studies investigating clinical utility [15]. Reid et al. (2018) [16] in a review of pain measurement in cats and dogs stated that “an instrument lacking in utility is of little use in the clinical arena”. In the current study, the comments reported such as “it was useful” and “straightforward to use” suggest that the BRPS is a scale that, amongst other qualities, could be a practically useful within a clinical environment. There is evidence that compliance in using pain scales is often poor in veterinary medicine [17, 18] and a practical and easy-to-use tool such as the BRPS might be implemented more often, thereby improving the health and welfare of rabbits experiencing pain. Other comments given by the respondents during the clinical utility test such as “it is not clear when action is required” or “what score would prompt analgesia to be given” highlight the need of a cut-off point in the scale that would guide analgesic treatments. Therefore, further research should be carried out to determine the cut of point of the BRPS for rescue analgesia.

The construct validity of the BRPS was measured by comparing pain scores assigned to rabbits before and after elective surgery. This was based on two assumptions. The first one was that rabbits admitted for surgical procedures planned in advance such as neutering are pain free, this was confirmed by a pre-operative health check. The second assumption was that despite the analgesia given during the perioperative period, the animal will still show a degree of pain after surgery. We found that there was a statistically significant difference in pain scores for videos pre and post operative. Similarly, using the Italian version of the UNESP-Botucatu Unidimensional Composite Pain Scale to assess post-operative pain in cattle [19], the authors found a significant increase in pain scores after surgery when compared to the pre-operative period. In the CMPS for acute pain in cats (CMPS-F) [2], the ability of the scale to distinguish changes in pain before and after analgesia was tested. However, clinical significance was not reached, and this was attributed by the authors to the small number of animals studied. To assess the construct validity of the CANCRS, the authors implemented a different approach; comparing how frequently the pain scale scores matched the perceived pain of common conditions and concluded that the CANCRS was able to assess rabbits without pain, from those with discomfort and with moderate pain. Severe pain was not detected.

In the present study, the criterion validity was tested by comparing the BRPS to a numerical rating scale (NRS) and showed high correlation demonstrating the ability of the BRPS to detect and quantify pain similarly to other pain scales. A gold standard for comparison to the BRPS has not yet been recognised in this field. The RbtGS, a pain scale based on facial indicators [20], was for many years the only pain scale available for this species but only recently was tested for validity in a clinical environment [4]. Similarly, the CANCRS has only recently been developed and validated [4]. Due to the lack of an optimum pain scoring tool when the current study was carried out, the NRS was then considered as gold standard. Similarly, the NRS has been used to validate other multidimensional pain scales such as the Glasgow Composite Pain Scale in dogs [21], the CMPS for acute pain in cats (CMPS-F) [2] and the Italian version of the UNESP-Botucatu pain scale in cattle [19]. In contrast, a visual analogue scale (VAS) was compared to the CANCRS when discriminating pain levels in rabbits [4]. The results suggested that CANCRS performed better than the VAS at identifying animals in pain. The authors stated that the reliability and responsiveness to changes in pain levels of the VAS should be further researched. In 2001, Holton et al. [3] reported that a NRS is a more appropriate scale for assessing pain in companion animals such as in dogs when compared to a VAS. Nevertheless, both NRS and VAS are unidimensional scales and it has been suggested that they are more subjective than multidimensional pain scales when assessing pain as they require the observer to prioritise the clinical signs they utilise when scoring and they do not take in account the emotional component of pain in the animal [22].

The measurement of reliability is a common test often carried out in both human and veterinary medicine. It ensures that a pain assessment tool can provide reliable information even if used by several observers and over time. This is important because it ensures that the patient is properly assessed over time even in a busy clinical environment when several members of the staff are involved in the patient care.

Reliability is generally tested using statistical tests such as Cohen's kappa coefficient (κ) or intraclass correlation coefficients (ICC) [23]. The choice of test is usually based on the number of raters (two or more) and the type of variables (categorical vs continuous) that are analysed [23]. Recently, the Krippendorf coefficient alpha, the test used in the present study, was found to have more advantages compared to the other two tests. For example, it does not present limitations when testing more than two raters nor several categories of a measuring scale, and it shows good reliability both within and between raters. It also takes in consideration missing values, a limiting factor often seen with other tests [24]. These specific characteristics of the Krippendorf test ensured that the BRPS was adequately assessed for reliability. It is difficult to compare the current study with other studies as this is the first time that this test has been applied to a veterinary pain tool. This is unsurprising as in a recent review the use of Kappa and Krippendorf’s alpha test in epidemiological studies, the Krippendorf alpha test was used only 35 times compared to the 11,207 times for the Kappa coefficient [24]. This could be considered a limitation of the current study as it does not allow the comparison between studies and reduced the changes of identify research patterns.

### Limitations

Some limitations have been taken in consideration in this study. The number of respondents in the clinical utility tests may be considered small [25]. When carrying out a survey, an adequate sample size is paramount to be able to draw appropriate conclusions. Several methods can be used to calculate the sample size including using references of previous peer-reviewed papers and subject-to-item ratio [25]. However, a consensus on the ideal sample size in surveys has yet to be reached [25]. In this study, the sample size was calculated using subject-to-item ratio and the minimum number that was needed for statistical analysis was identified and reached. Moreover, the number of female veterinary staff compared to males was much higher and this could have an impact on the way the questionnaire was answered and on the results of the clinical utility tests.

Another limitation of this study was that the participants for the validity and reliability test were all female veterinary nurses. Previous studies suggest that females may have a different way of feeling and reporting pain compared to male colleagues [18, 26] and this could have had an impact on the scoring of the video clips during the current study. Moreover, pain assessment in a veterinary environment is generally carried out by veterinary nurses or veterinary surgeons and veterinary nurses together [17, 27]. A recent survey showed that, in the UK, female veterinary nurses comprise 97.3% of the veterinary nursing population [28]. Therefore, the participants of this study closely represented a clinical veterinary environment where female veterinary nurses are more likely to assess rabbit patients for pain. However, further research should be carried out to include veterinary surgeons and males in the assessment of pain in rabbits.

## Conclusion

In conclusion, the BRPS is a valid and reliable multidimensional pain assessment tool, with potentially great value for improving the management of rabbit acute pain in a veterinary setting. Its clinical utility was demonstrated using a survey approach, which to the authors’ knowledge, was one of the first carried out in veterinary medicine. The consensus of the respondents was that the BRPS is an easy and practical instrument that can help in the decision making on a daily basis. The BRPS was also tested for construct and criterion validity, and both were found to be highly statistically significant in distinguishing between animals with and without pain and quantifying different intensities of pain. The reliability of the BRPS was also demonstrated and the results showed that the BRPS can be used by different observers and over time.

The results of the present study show that rabbits can be objectively and accurately assessed for pain and that the BRPS has the potential to guide the administration of analgesic treatment, if necessary, although this is an aspect that needs further research.

## Methods

This study was approved by the Faculty of Health Sciences Research Ethics Committee (FREC-66205) of the University of Bristol. All the respondents and participants to this study gave a written informed consent. Moreover, all methods of this study were carried out in accordance with relevant guidelines and regulations. The study is reported in accordance with ARRIVE guidelines.

### Clinical utility test

Veterinary surgeons and nurses (respondents) working with rabbits in the United Kingdom (UK) were contacted via email in November 2019. They were provided with the novel BRSP and asked to assess up to four rabbits in acute pain using the novel pain scale, and to record the average time required to assess the rabbits. They were then asked to complete a questionnaire of ten questions using a five-point Likert scale ranging from one (strongly disagree) to five (strongly agree) (Table 2). The respondents were also asked to give general comments regarding the use of the novel scale.

To estimate the number of respondents necessary for this study, a subject to item (number of respondents to the total number of questions in the questionnaire) ratio of 1:2 to 1:10 [24, 29] was considered indicating a minimum sample size of 20 respondents.

### Validity and reliability

#### Video clips

A total of twenty-eight individually housed rabbits undergoing elective procedures (ovariohysterectomy-OVH and orchiectomy-OR) from four veterinary clinics in the South-West of the United Kingdom (UK) were included. They were video recorded (September 2018—March 2019) during the perioperative period from the moment they were hospitalised until the time they were discharged on the same day. The videos were recorded using a GoPro Hero7 Black® as described in Benato et al. (2021)(16).The inclusion criteria were rabbits of any age, sex and breed that were booked for one of the two elective surgeries: OVH, or OR. The owner had given informed consent for the videos to be recorded. Anaesthesia and analgesia were provided to the rabbits according to the normal protocol used at each of the four veterinary clinics(16). The most common protocol included induction with a combination of ketamine (5 mg/kg), medetomidine (0.1 mg/kg) and buprenorphine (0.05 mg/kg) and maintenance with isoflurane once the patient’s airways were secured. Meloxicam was given at 0.6 mg/kg.

For the purposes of this study, one continuous segment of five-minutes (video clip) was selected per rabbit before (60-min post admission) and after surgery (At 150-min of the recovery time). A total of 20 rabbits providing 40 video clips (Before *n* = 20; After *n* = 20) were then selected. Twenty rabbits were deemed sufficient for statistical analysis to hypothetically represent each point of the total score of the BRPS (0–18) and it was deemed likely that each point (0–3) for the six categories would be represented. The video clips were also selected based on the quality of the video footage.

#### Participants

Nine female veterinary nurse students (participants; all different individuals to those used during the clinical utility test) in their final year of their degree in BSc Veterinary Nursing and Companion Animal Behaviour at the University of Bristol were recruited for this study between February and April 2020. The participants were offered a small financial compensation at the completion of the study that consisted of three sessions with a gap of two weeks in between each session. The time between sessions was chosen to ensure the participants were not influenced by the previous session and to prevent any carryover effects.

The participants were asked to watch the video clips in the order given, and to score the rabbits either using the BRPS giving a total score of 0–18 (Day 1) or the NRS with a total score of 0–10 (Day 14). One participant withdrew from the study after 14 days leaving eight participants. The participants had three minutes to observe the rabbit in the video clip and extra two minutes, if they needed, to score the animal. The order of the forty video clips was randomised for each participant in each session using a computer based random order generator. This was to prevent anticipation of the scores based on the participants’ knowledge of the order of the video clips previously assessed. The participants were informed that the video clips were of rabbits recorded during the perioperative period but were blind to whether the rabbits were before or after surgery. Prior to the trial, the participants were trained by the researcher (LB) on how to use both the BRPS and the NRS using four video clips of rabbits with (*n* = 2) and without pain (*n* = 2) that were not included in the selected video clips. The participants scored the video clips alone in a room during a single session and hence were independent of one another.

#### Validity

The construct validity of the BRPS was measured by comparing the pain scores given by the nine participants scoring the video clips of the same 20 rabbits before and after surgery on Day 1. The content validity was tested comparing the scores given by the participants *n* = 8) using the BRPS and 14 using the NRS,

#### Reliability

Inter-rater reliability was tested evaluating the scores given by all nine participants for 20 rabbits on Day 1 while intra-rater reliability was assessed by comparing the total score given by each participant (*n* = 8) of the same video clips on Day 1 and Day 28.

### Statistical analysis

The data were saved on an excel spreadsheet (Microsoft Office 365 ®) and the statistical analysis carried out using IBM SPSS Statistics 26. The internal consistency of the clinical utility questionnaire was tested using Cronbach’s alpha test. An acceptable value of alpha was considered between 0.70–0.95 [14]. Descriptive analysis was used to describe the results of the clinical utility tests and reported as median values and range. The comments added by the respondents were saved on a word document (Microsoft Office 365®) and used for descriptive analysis.

Data normality was tested by visual examination of histograms and normal probability plots (P-P plot). Since the data did not show a normal distribution, non-parametric tests were used to test the validity of the BRPS. The construct validity was measured by comparing the score given by the participants before and after surgery using a Wilcoxon signed rank test. The criterion validity was tested using Spearman’s rank correlation test comparing the scores given using the BRPS and the NRS. Statistical significance was considered *p* < 0.05. Both inter- and intra rater reliability were tested using the Krippendorff’s alpha test. Values > 0.8 signified strong reliability [30].

## Data Availability

The datasets generated and/or analysed during the current study are not publicly available due confidentiality agreement but are available from the corresponding author on reasonable request.
